# On the Relations of Diseases of the Skin to Internal Disorders

**Published:** 1906-06

**Authors:** 


**On the Relations of Diseases of the Skin to Internal Disorders.** By L. Duncan Bulkley, A.M., M.D. Pp. xv., 175. London: Rebman Limited. 1906.

This book is a compilation of lectures delivered to physicians at the New York Skin and Cancer Hospital last year. It has for its apology a quotation from the *British Medical Journal* of January 22nd, 1870, expressive of the need for the study of skin diseases in their relations to the disorders of other organs. The book might be described as a compendium of reminders that the skin must not be considered as a separate organ unconnected with the rest of the economy. In this intention it succeeds, and the lesson requires learning. No one liveth unto himself, and no disease of the skin, even though parasitic, is independent of the condition of health, or the reverse, of the host. Even the flea will not annoy every man, while it is a recognised fact that ringworm, so infective in childhood, dies out at puberty. To understand these things it is necessary to have a far deeper knowledge of the chemistry of the body than we yet possess. There is no division of medical investigation which can be regarded as not bearing upon the subject by any dermatologist who aims at success. It is not sufficient to treat the disease, the patient has to be considered, understood, and treated effectively. The skin frequently serves as a visible picture of pathological processes. That these have been misunderstood in the past is not surprising, but with the advent of more exact science the swing of the pendulum from "disorder of the blood" to a "microbic causation" will come to rest at the action and interaction of the external and internal processes of the body with one another.

Although the text of the book is in 1870, the application is in the present, and the "finally" is in the future. The definition quoted, that a good dermatologist is "one who understands the skin thoroughly and everything inside it," involves a wide scope for dermatology, and no one would say "too wide."

